# Reply to Füller et al.: Exome-wide genetic associations with socioeconomic status and their pleiotropy among health outcomes

**DOI:** 10.1073/pnas.2508030122

**Published:** 2025-05-16

**Authors:** Xin-Rui Wu, Liu Yang, Bang-Sheng Wu, Wei Cheng, Jin-Tai Yu

**Affiliations:** ^a^Department of Neurology and National Center for Neurological Disorders, Huashan Hospital, Fudan University, Shanghai 200040, China; ^b^State Key Laboratory of Medical Neurobiology and Ministry of Education Frontiers Center for Brain Science, Fudan University, Shanghai 200040, China; ^c^Institute of Science and Technology for Brain-Inspired Intelligence, Fudan University, Shanghai 200433, China

We thank Füller et al. for their comments (1) on our publication investigating exome-wide genetic associations with socioeconomic status (SES) and their implications for health outcomes (2) and appreciate the opportunity to expand discourse on certain aspects of our findings.

First, we agree that SES plays a vital part in the burden of coronary heart disease (CHD) and cancer, as supported by compelling epidemiological research evidence (3–5). We selectively included 44 diseases (excluding cancer, which is a limitation to be noted) in the first part of phenome-wide association analysis (PheWAS) and identified nominally significant associations (*P* < 0.05) between the SES-associated variants and CHD. Specifically, rare coding variant aggregates in *EP400* and *ANKRD12* showed suggestive associations with chronic ischemic heart disease, and single common variants analysis identified 15 loci (e.g., rs1801265) associated with chronic ischemic heart disease and 10 loci (e.g., rs1881194) with myocardial infarction. Furthermore, SES-associated variants were linked to several known CHD risk factors and complications including atrial fibrillation/flutter, heart failure, atherosclerosis, and hypertension. Although hyperlipoproteinemia mentioned by Füller et al. (1) was not analyzed in the original article, we observed significant associations with quantitative blood lipid markers, including *GIGYF1* loss-of-function with low-density lipoprotein (LDL) [reported as a novel finding in a UK Biobank exome study focusing on cardiometabolic diseases and traits (6)] and common loci rs1367083 with high-density lipoprotein (HDL). The details of PheWAS results could be found in datasets S4–S9 in the original article (2). These genetic findings consistently suggest the pleiotropic effects of SES-associated variants on CHD and related traits.

A follow-up question is how to interpret the observed pleiotropy. The existing biological correlates of low SES discussed by Füller et al. (1), such as inflammation and epigenetic changes (7), provides helpful explanatory clues. However, the specific directions of these pleiotropic effects of individual genetic signals, and the pathways linking genetic factors, SES and health outcomes at the genome-wide level, remain an interesting question requiring systematic and in-depth investigation. Over the past decade of genome-wide association studies, summary statistics of common variants have been fruitfully leveraged through tools like polygenic risk scores and Mendelian randomization (MR). For instance, Wang et al. (8) performed two-sample MR analyses to assess the causal impact of SES indicators on longevity outcomes and inquiry of the mediating roles of common lifestyle factors and diseases. Similar analytical frameworks could be extended to rare variants. Clarifying the directionality of genetic effects would further guide the exploration of underlying biological mechanisms underlying SES-health gradient.

Finally, we emphasize that gene–environment correlations arise from multifactorial sources, including population stratification, family environments, and geographic regions (9). The Füller et al.’s suggestion that SES-associated variants may have regional prevalence (1) aligns with active process of gene-environment correlations, where genetic predispositions could influence migration and environmental selection (10). Regarding geographic indicators, while Middle Layer Super Output Areas that used in the original article were divided to be as homogeneous as possible in terms of household tenure and dwelling type (11), social heterogeneity inevitably persists. Future research could use finer-grained geographic divisions to improve the estimation of gene–environment correlations.

## References

[1] D. Füller, B. Sasko, O. Ritter, Genetic associations with socioeconomic status are limited in explaining the socioeconomic health gradient. Proc. Natl. Acad. Sci. U.S.A. **122**, e2505061122 (2025).40378010 10.1073/pnas.2505061122

[2] X. R. Wu , Exome sequencing identifies genes for socioeconomic status in 350,770 individuals. Proc. Natl. Acad. Sci. U.S.A. **122**, e2414018122 (2025). 39772748 10.1073/pnas.2414018122 PMC11745334

[3] W. M. Schultz , Socioeconomic status and cardiovascular outcomes: Challenges and interventions. Circulation **137**, 2166–2178 (2018). 29760227 10.1161/CIRCULATIONAHA.117.029652 PMC5958918

[4] S. Li , An umbrella review of socioeconomic status and cancer. Nat. Commun. **15**, 9993 (2024). 39557933 10.1038/s41467-024-54444-2 PMC11574020

[5] R. Hamad , Association of low socioeconomic status with premature coronary heart disease in US adults. JAMA Cardiol. **5**, 899–908 (2020). 32459344 10.1001/jamacardio.2020.1458 PMC7254448

[6] S. J. Jurgens , Analysis of rare genetic variation underlying cardiometabolic diseases and traits among 200,000 individuals in the UK Biobank. Nat. Genet. **54**, 240–250 (2022). 35177841 10.1038/s41588-021-01011-w PMC8930703

[7] Y. Baumer, T. M. Powell-Wiley, Interdisciplinary approaches are fundamental to decode the biology of adversity. Cell **184**, 2797–2801 (2021). 34048701 10.1016/j.cell.2021.04.010 PMC8493648

[8] C. J. Ye , Mendelian randomization evidence for the causal effects of socio-economic inequality on human longevity among Europeans. Nat. Hum. Behav. **7**, 1357–1370 (2023). 37386110 10.1038/s41562-023-01646-1

[9] A. Abdellaoui, C. V. Dolan, K. J. H. Verweij, M. G. Nivard, Gene-environment correlations across geographic regions affect genome-wide association studies. Nat. Genet. **54**, 1345–1354 (2022). 35995948 10.1038/s41588-022-01158-0 PMC9470533

[10] A. Abdellaoui , Genetic correlates of social stratification in Great Britain. Nat. Hum. Behav. **3**, 1332–1342 (2019). 31636407 10.1038/s41562-019-0757-5

[11] Office for National Statistics, Census geography. (2021). https://www.ons.gov.uk/methodology/geography/ukgeographies/censusgeography/.

